# The Causes and Factors Associated with Infant Mortality Rate in Ethiopia: The Application of Structural Equation Modelling

**DOI:** 10.3390/children10020397

**Published:** 2023-02-17

**Authors:** Endeshaw Assefa Derso, Maria Gabriella Campolo, Angela Alibrandi

**Affiliations:** Department of Economics, University of Messina, 98122 Messina, Italy

**Keywords:** infant mortality rate, Ethiopia, path analysis, structural equation model, standardise estimate

## Abstract

Infant mortality rate is a proxy measure of population health. Previous studies on the infant mortality rate in Ethiopia did not consider measurement errors in the measured variables and had a one-directional effect; little emphasis was placed on testing multiple causal paths at the same time. We used structural equation modelling for a better understanding of the direct, indirect, and total effects among causal variables in a single model. A path analysis was part of an algorithm providing equations that were relating the variances and covariances of the indicators. From the results, the maternal mortality ratio (MMR) was significantly mediating the influence of out-of-pocket expenditure (OOP) on infant mortality rate (IMR), and the fertility rate (FR) was significantly mediating the influence of GDP to IMR (β = 1.168, *p* < 0.001). The GDP affects the IMR directly and indirectly while the OOP affects IMR indirectly. This study showed that there was a causal linkage between the World Bank Health and Population Variables for causing IMR in Ethiopia. The MMR and FR were found to be the intermediate indicators in this study. Through the indicators, FR had the highest standardised coefficients for increasing the IMR. We recommended that the existing interventions to reduce IMR be strengthened.

## 1. Introduction

The infant mortality rate (IMR) is the death occurrence between birth and exactly one year of age per 1000 births [1] and has been regarded highly as a signal for the measure of population healthiness [2]. The IMR remains a representative measure of population health, a symbolic benchmark of a society’s overall robustness [3,4], and recent studies emphasize the health inequities experienced by this population that have effects on infant mortality and morbidity [5].

Nowadays, the infant mortality rate has decreased across countries inhabiting different positions in the world. However, considerable cross-national variation in infant mortality remains at the beginning of the twenty-first century [6,7] and child mortality reduction goals under the United Nations Millennium Development Goals (UN MDGs) has not been achieved [8]. UN member states, instead of MDGs, set out Sustainable Development Goals (SDGs) in 2015 [9] as part of the 2030 agenda to end preventable deaths of newborns and children under 5 years of age, with all countries directed to reduce the neonatal mortality to at least as low as 12 per 1000 live births and under −5 mortality to at least as low as 25 per 1000 live births (SDG 3.2). Despite that, overall actions to meet the goals is not yet advancing at the speed or scale required [10].

The UN in 2018 rated that 6.2 million children and adolescents under the age of 15 years died from preventable causes. Among these deaths, 5.3 million occurred in the first 5 years and half of these in first month of life. Despite that the burden of those deaths was decreasing globally, Sub-Sahara Africa and South Asia account for the maximum proportion of child deaths. Four out of every five deaths of children under the age of five occur in these regions. Children in Sub-Saharan Africa are more than 15 times more likely to die before the age of 5 than children in the highly developed world. In Ethiopia, the IMR was 77 in year 2005 and it was 59 in 2011 per 1000 live births [11]. The country’s IMR declined from 97 per 1000 live births in year 2000 to 59 in year 2011, and neonatal deaths per 1000 live births showed a decline over time from 54 in year 1990 to 37 in year 2011, but it was unlikely that the MDG target of 31 per 1000 live births was achieved in the year 2015 [12].

There have been different factors that contribute to death in countries which have a high infant mortality. Some of the factors are malaria, malnutrition, lack of infrastructures, poverty, and poor health facilities [13]. High infant mortality signifies demographic and socioeconomic exposures and morbidity during pregnancy [14].

Scholars confirm that there were different predictors of IMR. A study conducted in African countries in 2014 revealed that the fertility rate, domestic general government health expenditure, and GDP per capita were found to be significant predictors of infant mortality [15].

In addition, fertility and GDP per capita were the most influential variables of the infant mortality rate among all the explanatory variables used in the analysis. Real GDP has a negative relationship with fertility and on the other hand, fertility is positively correlated with IMR [16,17]. Factors such as the Bolsa Família Program (BFP), per capita income, and fertility rate are associated with infant deaths [18,19,20]. Fertility appeared to influence infant mortality and it significantly affects the infant mortality rate in a positive way [21]. In low-income countries with minimal access to medical services and short intervals between births increases the infant mortality risk about fourfold [22,23]. A woman with high fertility setting has a greater risk of maternal death than in low fertility settings [24] and the maternal mortality ratio has been strongly associated with infant mortality [14]. Maternal mortality (in obstetric complications, obstructed labour, and hemorrhage) can put neonates at an increased rate of death [25] and maternal and infant mortality was closely linked to and responded in a similar manner to the same social, economic, and medical determinant of mortality rates [26]. Analogous to the maternal mortality ratio, the risk of maternal death varies largely across countries. Women in Sub-Saharan Africa have the highest risk of maternal death (1 in 38), followed by South Asia (1 in 240) [27]. Contemporarily, in order to prevent children’s deaths, efforts targeting maternal mortality must address inequalities in the access to care at the community, facility, and policy level [28].

Out-of-pocket (OOP) health expenditure significantly reduces maternal health, as it leads to a decrease in the skilled birth attendance by increasing the maternal mortality ratio [29,30]. The population in low-income countries is often exposed to out-of-pocket (OOP) and related indirect costs for their illnesses for health care, and this infers that the household’s health expenditure reduces the infant and maternal mortality across low-income countries to reach a goal of ensuring healthy lives and people’s well-being [31]. Moreover, according to the study conducted by [32], higher government spending on health services can be shown to provide better overall health results for children and in turn it reduces the infant mortality rate.

The Bacillus Calmette-Guerin (BCG) vaccine is given soon after birth to infants to decrease the incidence of tuberculosis (TB) disease and TB-associated mortality in childhood [33,34]. The lack of BCG vaccination in the first week of life was highly associated with the infant mortality rate [35]. The WHO currently suggests the BCG vaccination at birth for developing countries except for preterm infants who should be vaccinated when they reach the age of 40 weeks [36]. The infant mortality rate was lower for BCG vaccinated than for unvaccinated [37].

Accordingly, the IMR in Ethiopia could be attributed to many different factors [38,39,40,41,42,43,44]. Previous studies have mostly employed only observed variables (variables that are measured in data collection processes) and a one-directional effect to discover relationships in the data set through a difference-in-differences (Diff-in-Diff) analysis, spatial patterns of infant mortality, multiple linear regression and/or correlation analyses, multiple logistic analyses, and other multivariate statistical models to explore the factors associated with IMR. Furthermore, research conducted in Egypt on the infant mortality rate [45] used structural equation modelling based on economic indicators, and this study passed over the most influential variables mediating variables, model identification and validation, which are the basic determinants for structural equation modelling.

In this paper, we examined the association of IMR in Ethiopia between 2000 and 2019 based on the World Bank Health Nutrition and Population Statistics variables. We used structural equation modelling (SEM), multivariate statistical methods, for better understanding the direct, indirect, and total effect of the given variables. This approach improved the understanding of mechanisms of the relationships among various factors and allowed us to test the research hypotheses in a single process by modelling complex relationships among many observed and latent variables [46,47]. The SEM or analysis of covariance structure is a confirmatory approach, dealing with measurement errors in observed variables and is more suitable for testing the hypothesis than other multivariate statistical methods. Most of the statistical methods other than structural equation modelling try to discover relationships through the data set. However, SEM asserts the correspondence of the data of the relations in theoretical model [48,49].

In a recent commentary, scholars expressed concern about the scarcity of SEM models in epidemiological research even if there was the availability of user-friendly software (e.g., SPSS AMOS, EQS, Mplus) and urged epidemiologists to use SEM models more frequently [50,51,52]. The purpose of this study was to test and develop a hypothesised model for better understanding the direct, indirect, and total effects for the given variables on the infant mortality rate by estimating the parameters in the interest of obtaining a minimal residual covariance from the World Bank dataset between 2000 and 2019. We expect that the findings from our study will improve the planning and intervention to take measures for preventing infant mortality in Ethiopia.

**Figure 1:**
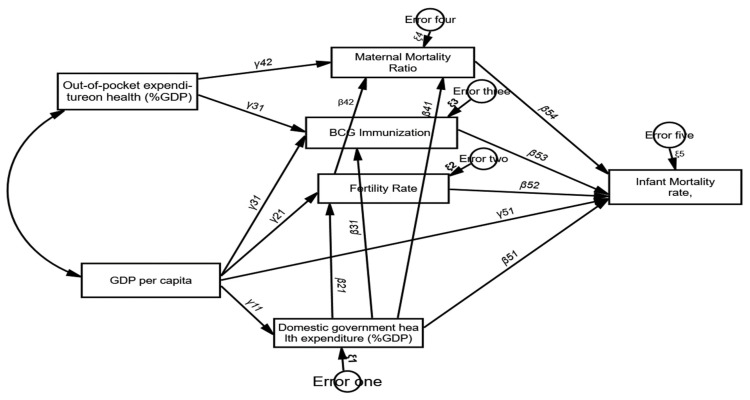
The directed cyclic graph/path diagram of the research model.

Basing on the previous studies, we developed the following hypotheses and the hypothesised value of each path is included in the following directed diagram (see Figure 1). The hypotheses of this study are stated as:

**H1.**  *There is a direct effect of out-of-pocket expenditure for health (% of GDP) on the maternal mortality ratio and Immunization (BCG).*

**H2.**  *Both BCG Immunization and the maternal mortality ratio mediate the influence of out-of-pocket expenditure on health (% of GDP) on the infant mortality rate.*

**H3.**  *The higher level of the fertility rate is associated with a higher level of the maternal mortality ratio.*

**H4.**  *Government health expenditure has a direct effect on the fertility rate, BCG immunization, maternal mortality ratio, and infant mortality rate.*

**H5.**  *GDP per capita has a direct effect on the domestic general government health expenditure (% of GDP), Immunization (BCG), fertility rate, and infant mortality rate.*

**H6.**  *Domestic general government health expenditure (% of GDP), fertility rate, and Immunization BCG mediate the influence of GDP on the infant mortality rate.*

## 2. Materials and Methods

Our analysis used pooled panel data from 2000 to 2019 from the World Bank Health Nutrition and Population Statistics. This dataset was from the data catalog of the World Bank which provides data on key health, nutrition, and population statistics gathered from international sources (such as the WHO). Some of the series included in this indicator were the population dynamics, nutrition, reproductive health, health financing, medical resources, immunization, infectious disease, HIV/AIDS, and population projection. Furthermore, based on the literature, we considered the GDP per capita, out-of-pocket expenditure on health, BCG immunization, maternal mortality ratio, fertility rate, domestic general government expenditure on health, and infant mortality rate for testing several causal paths simultaneously over 20 years (2000–2019) in Ethiopia. Analyses were performed using SPSS AMOS and STATA 14. The dataset we used is freely available https://data.worldbank.org/ (accessed on 4 October 2022).

The variables considered in SEM are called either endogenous or exogenous variable [53].

Moreover, these endogenous and exogenous variables can be illustrated through the arrows that come out of or go into each rectangle [54].

The exogenous variables considered in this study were the GDP per capita and out-of-pocket expenditure on health (% GDP).

The endogenous variables were the BCG immunization, maternal mortality ratio, fertility rate, domestic general government expenditure on health (as a share of GDP), and infant mortality rate. In the following table (Table 1) we report in more detail the variables considered.

### 2.1. Statistical Model

We used this multivariate method for the causal correlation among two or more variables and tested the essential theory from empirical data. To being thought of as a form of SEM focusing on causality, the path analysis describes the direct dependence among a set of variables. SEM is carried out by the graphical relationship and numerical result accordingly.

#### 2.1.1. Path Analysis

The path Analysis represents a methodological improvement regarding multivariate techniques used in modelling indicators and it allows the investigation of more complex models [55]. Furthermore, the path analysis rules of Wright [52] involve tracing paths in the graph as part of an algorithm giving equations relating the variances and covariances of the indicators and it is represented by a diagram, called a directed graph (path diagram). In directed graphs, the vertices represent continuous variables, edges represent some notion of correlation and causation, and the relations in the diagram are the parameters of the equations to be estimated, called path coefficients, presenting the responses of endogenous variables to other endogenous or exogenous variables, while other variables in the model are held constant [52,56].

Each node in the path analysis was defined by the variables *y*_1_ … *y_n_* and there was a directed edge from *y*_*i*_ to *y_j_* if the coefficient of *y_i_* in the equation for *y_j_* was distinct from zero [57]. Moreover, there was a mediation where one variable (exogenous) caused variation in another variable (endogenous), and the mediator hypothesis was supported if the variables BCGI, MMR, FR, and GGHE-D were significant.

From Figure 1, all indicators were represented by rectangles, it indicated that there was no latent variable in the model, and all arrows flowed one way with no feedback looping (recursive model). The measurement errors for the endogenous variables were uncorrelated [58,59]. Our directed graph set out all the causal linkages between variables to evaluate the possible hypothesis and $\beta_{ij}\;\mathrm{and}\;\gamma_{ij}\;\mathrm{were}$ the coefficients. This is illustrated in the following figure (Figure 1).

#### 2.1.2. Structural Equation Model (SEM)

In SEM, a series of endogenous variables are related to each other as well as to a series of exogenous variables. This model has three major advantages over traditional multivariate techniques: (1) the explicit assessment of measurement error; (2) the estimation of latent (unobserved) variables via observed variables; and (3) model testing where a structure can be imposed and assessed as a fit of the data [60,61,62].

Thus, to examine the linear causal relationships among variables, we used SEM and the specification of the model was follows.

Let: y be an p × 1 vector of endogenous variables, x is a q × 1 vector of exogenous variables, β_p×p_ gives the regression coefficients of endogenous (y) variables on other endogenous variables (it is the matrix of β′ regression path coefficients between endogenous to endogenous), γ_p×q_ gives the regression coefficients of the exogenous variables (x) on endogenous variables (y) whose *i*th row indicates the endogenous variable and the jth column indicates the exogenous variable, and $\varsigma_{px1}$ is the vector of errors in the equations (i.e., regression residuals) as a vector of the model errors associated with each endogenous variable. The variances and covariances of the endogenous variables are modelled as a function of the exogenous variables. Then, the general form of a SEM path analysis model is expressed in the matrix equation:

y = *β*y + *γ*x + *ς*

(1) $$y={\left(I-\beta \right)}^{-1}\gamma x+{\left(I-\beta \right)}^{-1}\;\varsigma$$

Then the variance of the endogenous variables (y variables) is:

$$V(y)=E({\mathrm{yy}}^{\prime })=E\;[({\left(I-\beta \right)}^{-1}\gamma x+{\left(I-\beta \right)}^{-1}\;\varsigma )\;((I-\beta {)}^{-1}\gamma x\;+{\left(I-\beta {)}^{-1}\;\varsigma \right)}^{\prime })]$$

(2) $$\sum =(I-\beta {)}^{-1})[\gamma \Phi \gamma^{\prime }+\psi ]{\left(I-\beta^{\prime }\right)}^{-1}$$

provided that the variances of the exogenous variable x are defined as:

V(x) = E(xx′) = Φ, and V (*ς*) = E (*ς*′) = *ψ*

Similarly, the covariance between the exogenous variable, x and the endogenous variables (y variables) (covariance between x and y) is:

Cov(x,y) = E(xy′) = E[x((I − *β*)^−1^*γ*x + (I − *β*)^−1^
*ς*′)] (3)

$$\sum =\Phi \gamma^{\prime }{\left(I-\beta^{\prime }\right)}^{-1}$$

Assumptions:

ς is uncorrelated with x, cov (ς,x) = 0,

$$E\left(\varsigma \right)=0$$

|I − β| ≠ 0, is invertible, (I ≠ β),

E(x) = E(y) = 0

Therefore, putting all the variance–covariance together,

(4) $$\sum =\left[\begin{array}{cc} \sum_{\mathrm{yy}} & -\\ \sum_{\mathrm{xy}} & \sum_{\mathrm{xx}} \end{array}\right]$$

Here, x, y, and ς are Gaussian random vectors; x ~ N(μ_x_, ∑_x_); y ~ N(μ_y_, ∑_y_); the stochastic error has a multivariate Gaussian distribution which has for the mean a zero vector and for the covariance matrix a diagonal matrix where the diagonal elements are ψ11, ψ22, ψ33, ψ44, and ψ55 (i.e., ς ~ N(0, ψi). Furthermore, the variance–covariance of exogenous variables was determined outside of our model. The causality of infant mortality based on our variables expressed as a single matrix is:

$$\left[\begin{array}{c} \begin{array}{c} \mathrm{GGHE}-D\\ \mathrm{FR} \end{array}\\ \mathrm{BCG}\\ \mathrm{MMR}\\ \mathrm{IMR} \end{array}\;\right]=\left[\begin{array}{ccccc} 0 & 0 & 0 & 0 & 0\\ \beta_{21} & 0 & 0 & 0 & 0\\ \beta_{31} & 0 & 0 & 0 & 0\\ \beta_{41} & \beta_{42} & 0 & 0 & 0\\ \beta_{51} & \beta_{52} & \beta_{53} & \beta_{54} & 0 \end{array}\right]\left[\begin{array}{c} \begin{array}{c} \mathrm{GGHE}-D\\ \mathrm{FR} \end{array}\\ \mathrm{BCG}\\ \mathrm{MMR}\\ \mathrm{IMR} \end{array}\;\right]+\left[\begin{array}{c} \begin{array}{cc} \gamma_{11} & 0\\ \gamma_{21} & 0 \end{array}\\ \begin{array}{cc} \gamma_{31} & \gamma_{32}\\ 0 & \gamma_{42}\\ \;\gamma_{51} & 0 \end{array} \end{array}\right]\left[\begin{array}{c} GDP\\ OOP \end{array}\right]+\left[\begin{array}{c} \begin{array}{c} \varsigma_{1}\\ \varsigma_{2} \end{array}\\ \varsigma_{3}\;\\ \varsigma_{4}\;\\ \varsigma_{5} \end{array}\;\right]$$

By hypothesis, some of the elements of β and γ are fixed to zero and the zeros on the diagonal of β imply that a variable cannot cause itself.

The variance–covariance matrix of the exogenous variables used in the model were given by:

$$\Phi =\;\;\left[\begin{array}{cc} \mathrm{var}\left(\mathrm{GDP}\right) & -\\ \mathrm{Cov}\left(\mathrm{GDP},\mathrm{OOP}\right) & \mathrm{var}\left(\mathrm{OOP}\right) \end{array}\right]$$

Similarly, the variance–covariance matrix of the error terms ($\varsigma_{1}$, $\varsigma_{2},\varsigma_{3},\varsigma_{4},\;\mathrm{and}\;\varsigma_{5}$) is given by:

$$\psi i=\left[\begin{array}{ccccc} \psi 11 & 0 & 0 & 0 & 0\\ 0 & \psi 22 & 0 & 0 & 0\\ 0 & 0 & \psi 33 & 0 & 0\\ 0 & 0 & 0 & \psi 44 & 0\\ 0 & 0 & 0 & 0 & \psi 55 \end{array}\right]\;\;i=1,2,3,4,5$$

Typically, these variances and covariances of the exogenous variables x_1_ and x_2_ and the error terms of the error variances are free parameters, but the covariances of error variances are fixed to zero.

In SEM, each indicator should follow multivariate normality for each value of each other indicator and a maximum likelihood estimation (MLE) is the dominant method for estimating structure (path) coefficients [63].

If we have a p × 1 random vector X that is distributed according to a multivariate normal distribution with a population mean vector μ and population variance covariance matrix Σ, then this random vector, X, could have the joint density function in the expression of

ϕ(x)=(12π)p2Σ −12 exp {−12(x−μ)′Σ−1(x−μ)}, X~N (μ, Σ)

where $\left|\Sigma \right|$ is the determinant of the variance–covariance matrix Σ and $\Sigma^{-1}$ is the inverse of the variance–covariance matrix Σ.

Identification is the crucial problem when using SEM and no reliable quantitative conclusion can be derived from non-identified models. From the three categories of SEM based on their identification, in exact identified models with all variables interconnected, the parameters have an interpretation (Df = 0) while unidentified models lack sufficient information to yield a convergent solution of the parameter estimates (Df < 0). Moreover, an overidentified model contains too many restrictions for convergence and has more than enough information to obtain a meaningful estimate (Df > 0) [64,65,66].

For the path analysis model, let *P* be the total number of exogenous and endogenous variables in the model and let t be the number of the numbers of free parameters.

$$t\text{-}\mathrm{rule}=\frac{p\left(P+1\right)}{2}\geq t$$

The difference gives the number of degrees of freedom (Df) for the model:

(5) $$\mathrm{Df}=\frac{P\left(P+1\right)}{2}-t$$

The model fit statistics provide information about the goodness of fit indexes and their cut-off values for model evaluation. The more fit the indices applied to the SEM model are, the more likely that a misspecified model will be rejected [67,68].

Furthermore, the measures of the goodness of fit cut-off value for the Chi-square associated *p*-value (*p*) was ≥0.5 and the cut-off value for the Root Mean Square Error of Approximation (RMSEA) was 0.05 < value ≤ 0.08 [41]. Complementarily, 0.90 ≤ value < 0.95 is an acceptable cut-off value for the Comparative Fit Index (CFI) and Tucker–Lewis Index (TLI) [69].

## 3. Results

### 3.1. Descriptive Statistics

Descriptive statistics were used for summarizing the baseline characteristic of the population. As shown in the following table (Table 2), the mean number for the infant mortality rate was 58.16 per 1000 live births in the sample of 20 years for the World Bank data from 2000 to 2019 in Ethiopia. In our settings, the maximum number of infants dying before reaching one year of age was recorded in the year 2000 with a value of 87.2 per 1000 live births and the minimum value was in the year 2019 with a value of 36.6 per 1000 live births each year. The maximum values of the fertility rate were 6.543 births per woman in the year 2000 and 1030 was the maximum maternal mortality ratio encountered in the year 2000 per 100,000 live births. The mean number of public expenditures on health from domestic sources as a share of the economy as measured by GDP was 1.18 and the mean out-of-pocket expenditure, GDP per capita, and BCG immunization were given as 37.81, 67.8, and 395.23 respectively.

From Table 2 of the assessment of the normality column, the univariate critical values of both skewness and Kurtosis of the observed endogenous variables and exogenous variables lied between −1.96 and +1.96 (all these *p*-values are ≥0.05) and the critical value of the multivariate normality of the model was −0.191. We retained the null hypothesis and considered the sample as coming from a normal distribution.

### 3.2. Model Identification

We used this model identification to check whether the number of parameters to be estimated was greater than the number from unique information provided by the variance–covariances or not. From our model, we had 5 endogenous and 2 exogenous (7 rectangles from the path diagram depicted above). The covariance matrix was given by

$$\sum {}_{7*7}=\;\frac{7\left(7+1\right)}{2}=28\mathrm{variances}\;\mathrm{and}\;\mathrm{covariances}.$$

Complementarily, we had 22 free parameters (8 non-zero from β; 6 non-zero from γ, 3 variances/covariances in Φ from exogenous variables, and 5 residual variances in the diagonal of ψ). Therefore, the model degrees of freedom was (Df) = 28 – 22 = 6, so our model was overidentified, which was good because there were extra degrees of freedom to work with [65].

### 3.3. Path Analysis

**Figure 2:**
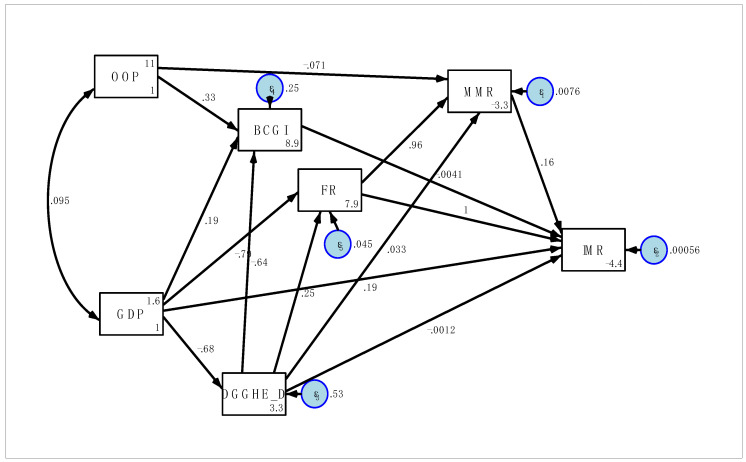
The path diagram: the path standardised coefficients of the risk factors on IMR (2000–2019).

In Figure 2, the directed graph was displayed for each variable to test the hypothesised. The path coefficients and errors presented in Figure 2 were standardised estimates and accordingly, the analysis was carried out in SPSS AMOS. The diagram shows how one variable was associated with a subsequent variable in the causal chain. The direct effects were dedicated to the straight influence of one variable on another observed variable without any mediation and the effects of more distant variables were mediated indirectly through intervening.

### 3.4. Structural Equation Model

Table 3 shows the values of the standardised parameter estimate (direct, indirect, and total effects) of the structural equation model by employing the maximum likelihood estimation which gathered the loadings for each variable of the model.

This study found evidence that the out-of-pocket expenditure (OOP) had direct effects on the maternal mortality ratio (β = −0.071, *p* = 0.003) and BCG immunization (β = 0.327, *p* = 0.024) and that as OOP increased by one unit, MMR decreased by 0.71 unit, and immunization (BCG) increase by 0.327 unit, while other variables were held constant. In addition, the coefficient for the maternal mortality ratio (MMR) was a statistically significant predictor of the infant mortality rate in Ethiopia with (β = 0.141, *p* = 0.009), while the coefficient of BCG immunization was insignificant for the infant mortality rate with (β = −0.0041, *p* = 0.774). Based on the loading and *p*-values (see in Table 3), the indirect path coefficient of the OOP to IMR through MMR was negative and significant (β = −0.012, *p* = 0.034). Thus, MMR was significantly mediating the influence of OOP on IMR and BCGI was not a mediator for OOP to IMR. In conclusion: H1: “there is a direct effect of the out-of-pocket expenditure on health (% GDP) on the BCG immunization and maternal mortality ratio” was fully supported and H2: “both the BCG Immunization and maternal mortality ratio mediate the influence of out-of-pocket expenditure on health (percentage of GDP) on the infant mortality rate” of the research hypothesis was partially supported.

Looking at the effects of GDP on the endogenous variables, GDP had a significant total effect on the fertility rate with (β = −0.959, *p* < 0.001), part of which (β = −0.175 and *p* = 0.004) was indirect through GGHE-D, and when GDP went up by 1 unit, FR went down by 0.175 unit due to the indirect (mediated) effect of GDP on FR in addition to any direct (unmediated) effect that GDP may have had on FR. GDP was also a significant predictor of the infant mortality rate (β = −0.94, *p* < 0.001) and government expenditure on health (β = −0.683, *p* < 0.001), respectively. The direct path coefficient from GDP to BCGI was insignificant (β = 0.188, *p* = 0.260). Moreover, as GDP increased by one unit, FR decreased by 0.959 units, the government expenditure on health decreased by 0.683 units, and IMR decreased from 0.941 units to 0.625, while other variables were held constant. The research hypothesis H5: “there is a direct effect of GDP on GGHE-D, BCGI, FR, and IMR” was partially supported.

Further, when we considered the direct effects of government expenditure on health for other endogenous variables, the path coefficient was negative and significant for BCGI (β = −0.640, *p* < 0.001), positive and significant for FR (β = 0.256, *p* < 0.0.001), and insignificant for MMR (β = 0.246, *p* = 0.386), respectively. The total effects of government expenditure on health (GGHE-D) on IMR was significant (β = 0.306, *p* = 0.017), part of which (β = 0.308, *p* < 0.001) was indirect through FR. There was also a significant effect of the fertility rate on maternal mortality ratio (β = 0.96, *p* < 0.001). In conclusion, H4: “there is a direct effect of GGHE-D on FR, BCGI, MMR, and the IMR” was partially supported and H3: “a higher level of FR is associated with a higher level of MMR” was supported.

Our model also revealed that there were direct positive effects between FR and IMR (β = 1.168, *p* < 0.001) and between MMR and IMR (β = 0.156, *p* = 0.009). The direct path coefficients from BCGI and GGHE-D to IMR were insignificant with the standardised beta coefficient and *p*-values of (β = −0.007, *p* = 0.774) and (β = −0.002, *p* = 0.915), respectively. Based on the loadings or standardised coefficients, the FR had the highest standard coefficients (β = 1.168, *p* < 0.001) for increasing the infant mortality rate (IMR), part of which was indirect through MMR (β = 0.136 and *p* = 0.009). As the fertility rate increased by one unit, the infant mortality rate increased by 1.168, through which 0.136 unit was indirect through the maternal mortality ratio while all other variables were held constant (Table 3).

In addition to the above established relationships of the variables in the model, structural relationships between the set of variables were taken into consideration. Table 4 represents the covariance of how much two variables move together. The relationship between MMR and IMR (Σ = 0.99), MMR and FR (Σ = 0.99), and MMR and GGHE-D (Σ = 0.79) was positive and increasing while the relationship between MMR and BCGI (Σ = −0.75), MMR and OOP (Σ = 0.17), and MMR and GDP (Σ = 0.96) was negative and decreasing (see Table 4). The value of the covariance did not give any more information further than the directionality [27].

### 3.5. Assessment of the Overall Goodness of Fit

The model summary (see Table 5) provided the equation-by-equation goodness of fit statistics for the endogenous variable, which was displayed by the equation level variance decomposition along with the coefficient of determination ($R^{2}$), Bentler-Raykov squared multiple correlation coefficient ${\left(mc\right)}^{2}$, and the correlation between them and their predictors (mc). The values of the coefficient of determination ($R^{2}$) and Bentler-Raykov squared multiple correlation coefficient ${\left(mc\right)}^{2}$ as measures of the goodness of fit statistics are equivalent in recursive structure equation modelling [53].

According to the results in Table 5 above, the correlation between MMR and its predictors was 0.996 and the variance of MMR explained by its predictors was 0.993 or 99.3% of the variation explained by MMR in the equation for the endogenous variable MMR. Similarly, the correlation between FR and its predictors was 0.978 and 95.5% of the data fit the model for the endogenous variable FR and the model equitation of the endogenous variable IMR explained 99.5% of the total variation of implied causality.

Further, because the χ^2^ goodness of fit criterion is very sensitive to the sample size, often other descriptive measures of fit are used in addition to the absolute χ^2^ test and there should be a combination of at least two goodness of fits [41,68]. The overall model fit for the structural equation model was adequate to good in terms of the CFI (0.932) and TLI (0.961).

Table 6 reveals the residual covariances (i.e., the difference between the sample covariances based on the sample data and the covariances implied by the fitted model) that provided a natural estimate of the fit of covariance structure models and this covariance residual value was smaller (all values were less than 1.96 in absolute value). The model was supported as the implied covariance matrix did not differ significantly from the empirical covariance matrix. This smaller value indicated the best fit of the covariance structure model. The larger in absolute value the residual covariance is, the worse the fit [70].

The results presented in Table 7 indicate the parameter estimation of coefficients of the observed variables, the standard error, significant values, and the 95% confidence interval for the final structural equation model for the infant mortality in Ethiopia. It revealed the direct effect of one endogenous or exogenous observed variable on another endogenous variable.

So based on Table 7 and Figure 1 the final structural equation model was:
GHE = −0.683 GDP + 0.5349707, *R*^2^ = 46.6%FR = 0.256 GGHE-D + −0.786 GDP + 0.0450516, *R*^2^ = 95.5%BCG = −0.639 GGHE-D + 0.327 OOP + 0.2451441, *R*^2^ = 75.5%MMR = 0.961FR + −0.071 OOP + 0.0075514, *R*^2^ = 99.3%IMR = +1.03FR + 0.156MMR + 0.192GDP + 0.0005578, *R*^2^ = 99.5%

## 4. Discussion

We used SEM to estimate the direct, indirect, and total effects of variables, to accredit the presence of connections between them, and test the hypothesised model based on World bank data on IMR. From a sample of 20 years of World Bank data, the occurrence of IMR was decreasing, which could be justified by the advancement of mother and childcare activity in Ethiopia. Although this represents an overall decline in the infant mortality between the year 2000 to the year 2019, Ethiopia accounts for the highest infant mortality rate, as it was reported at 35.4% in 2020 and the country did not achieve the extent of the sustainable development goals (SDGs) of target focuses on “ensuring healthy lives and promoting the wellbeing of for all” [13].

From the study using path analysis (directed graph) and structural equation modelling, we found that the variables MMR, FR, and GDP significantly affected the IMR directly. In addition, the indirect path coefficients from the OOP and FR to IMR through MMR and indirect path coefficients GGHE-D and GDP to IMR through FR were significant. However, the variable BCGI was not influential for IMR. Consequently, the FR and MMR were the mediating variables on IMR and among all variables that had an influence on IMR, FR had the highest standardised coefficient. Complementarily, the OOP and FR had an effect on MMR directly and the GDP and GGHE-D affected MMR indirectly through FR. Moreover, GGHE-D affected FR directly while GDP affected FR direct and indirectly. Contemporarily, as indicated by our results, government spending on health had a significant effect on reducing the infant mortality and its coefficient depended on the economic level of the country and the level of good governance. So, based on our study area Ethiopia, one of the low-income countries, reductions in government expenditures on health in the country were associated with a significant increase in the infant mortality rate. Our result was in accordance with [32], where higher government spending on health services can be shown to provide better overall health results for children and in turn reduces the infant mortality rate. In our analysis, residual covariances of this SEM were smaller (all values are less than 1.96 in absolute value). This smaller value indicated the best fit of the covariance structure model. The larger the absolute value the residual covariance was, the worse the fit [65].

There were significant direct effects of the OOP on MMR and BCGI. Moreover, the MMR was significantly mediating the influence of OOP on IMR, but there was no indirect effect of OOP on IMR through BCGI. Our result was in line with [25,26] that stated that the maternal and infant mortality was closely linked and responded in a similar manner to the same social, economic, and medical determinant of mortality rates. Ultimately, H1: “there is a direct effect of OOP on BCGI, and MMR” was fully supported while H2: “both BCGI and MMR mediate the influence of OOP on IMR” of the research hypothesis was partially supported. This finding is also in line with another previous study in Egypt [45]. Considering this result, BCGI was not significantly associated with IMR. Contrary to our results, the authors of [36] revealed that IMR was lower for BCGI vaccinated than unvaccinated. This variability could be better BCGI vaccination coverage in Ethiopia, as it was 56% in 2000 and 90.27% in 2019 [71].

Looking at the direct effects of GDP on other endogenous variables, GDP had was a significant and negative predictor of FR, part of which was indirect through GGHE-D, and this was in addition to any direct (unmediated) effect that GDP may have had on FR. This study was in accordance with the study conducted in Pacific Island countries [16] and the study from the developed world [17]. Our results in Ethiopia were entirely consistent with those from studies that observed that the GDP had a negative association with FR, and in return, the IMR was positively correlated with fertility [15,16,17]. Furthermore, FR was more likely to affect IMR. This result is also consistent with other studies [21,22,23]. This is because in the developing world, parents consider children as virility, and they used their children for work and to bring in an income to the family, and Ethiopia has a total fertility rate of 4.6 children per woman [72]. Lastly, our research hypothesis H5 was partially supported.

There had also been a significant effect of FR on MMR and this result was in line with the study conducted in Nepal [14]. In conclusion, H4: “there is a direct effect of government health expenditure on fertility rate, BCG immunization, maternal mortality ratio, and infant mortality rate” was partially supported and H3: “a higher level of fertility rate is associated with a higher level of maternal mortality ratio” was supported.

Our study encircled a configuration for the application of SEM to IMR and this analysis contributes to a growing body of literature supporting multiple hypotheses in the IMR World Bank Health and Nutrition indicators. We considered the simultaneous linkages of the World Bank Health Nutrition and Population Statistics variables on IMR. The results showed that the GDP and the intermediate variables, MMR and FR, where other observed variables affect IMR through them, were the pivotal observed variables that had a critical effect on IMR. Although a lot has been done to achieve the research objectives, there were some limitations and shortcomings. First, even though the model requires larger sample sizes and longer study periods for better accuracy, we could not find representative and enough data from the database based on the given indicators before 2000. Secondly, the research covered limited numbers of endogenous and exogenous variables. Thus, future researchers should consider more variables and examine different relationships between the cause of IMR and the government having to increase its incentives on health services to improve infant health.

## 5. Conclusions

We used a structural equation model to examine different connections between observed variables and to recognize the direct, indirect, and total effects of IMR based on Health Nutrition and Population Statistics indicators. This study found that the maternal mortality ratio, fertility rate, government expenditure on health, and GDP per capita do have a significant impact on the infant mortality rate in Ethiopia and the study showed that that there was a reverse association between IMR and GDP. However, the model showed that BCGI was insignificant to the IMR. As we observed in the present study, a reduction in the fertility rate, improvement in the general care of mothers, and increasing the per capita GDP of the country are the most important factors for decreasing IMR. The variables FR and MMR were the mediators from OOP to IMR and from GDP to IMR, respectively. FR had the highest standard coefficients for increasing the infant mortality rate (IMR) directly and indirectly through MMR. In line to this, both government and stockholders should design and implement programs to decrease the FR and MMR and increase the per capita GDP and OOP to decrease the rate of infant mortality. Therefore, from our research hypotheses, H1 and H3 are fully supported while the rest of the research hypotheses H2, H4, H5, and H6 were partially supported. From our model, the covariance residual value was smaller (all values were less than 1.96 in absolute value), and it showed a good estimate of the fit of covariance for structure models.

## Figures and Tables

**Table 1:** List of endogenous and exogenous variables and their abbreviation.

S. N	Observed Variables	Abbreviation	Definition
1	GDP per capita	GDP	Gross domestic product, the monitory wealth of the nation of one country’ goods and services over a given period, usually in one year.
2	Out-of-pocket expenditure on health.	OOP	Households or individual direct expenses to health institutions or health service providers (it does not include taxes and health insurances).
3	Domestic general government health expenditure on health (as % GDP)	GGHE-D	The share of current domestic government resources used to refund public health expenditure as a share of the economy, and it is measured by GDP.
4	Fertility rate	FR	The number of children born to a woman in her childbearing-age years and bearing children in accordance with the age-specific fertility rates of the specified year.
5	BCG immunization (% of one year-old children)	BCGI	A vaccine given to a one-year old who has received one dose of bacilli Calmette-Guerin expressed in a percentage.
6	Maternal mortality ratio	MMR	Annual number of female deaths per 100,000 live births from any cases (cases related to pregnancy).
7	Infant mortality rate	IMR	The probability of dying between birth and exactly one year of age per 1000 births.

**Table 2:** The descriptive statistics of the association of infant mortality in Ethiopia, the application of structural equation modelling path analysis (from year 2000 to year 2019).

	N	Minimum	Maximum	Mean	Std. Deviation	Assessment of Normality			
						Skewness	Critical Ratio	Kurtosis	Critical Ratio
Fertility rate	20	4.15	6.54	5.26	0.75	0.180	0.328	−1.186	−1.082
Out-of-pocket expenditure	20	31.34	46.54	37.81	0.607	1.108	−0.028	−0.026	0.607
Maternal Mortality ratio	20	354.00	1030.00	663.35	231.94	0.281	0.513	−1.380	−1.259
Infant mortality ratio	20	36.60	87.20	58.16	16.09	0.347	0.634	−1.134	−1.035
BCG Immunization	20	56.00	80.00	67.80	6.79	−0.332	−0.606	−832	−0.759
Gov.t expenditure on health	20	0.38	2.28	1.18	0.54	0.672	1.227	−0.606	−0.553
GDP per capita	20	111.93	855.76	395.23	251.41	0.447	0.815	−1.160	−1.059
Valid N (listwise)	20								
Multivariate								−0.960	−0.191

**Table 3:** The standardised paths for the direct, indirect, and total effects of each factor of the association of infant mortality in Ethiopia, (2000–2019).

Indexes/Pathway		Relation with Standardised Coefficients		
To	From	Direct	Indirect	Total
MMR <- OOP		−0.071 (*)	-	−0.071 (*)
MMR <- FR		0.96 (**)	-	0.96 (**)
MMR <- GGHE-D		0.246 (*p* = 0.386)	0.032 (**)	0.278 (**)
MMR <- GDP		-	−0.861 (**)	−0.861 (**)
BCGI <- OOP		0.327 (*)	-	0.327 (*)
BCGI <- GDP		0.188 (0.260)	0.437 (*)	0.625 (**)
BCGI <- GGHE-D		−0.640 (**)	-	−0.640 (**)
FR <- GGHE-D		0.256 (**)	-	0.256 (**)
FR <- GDP		−0.784 (**)	−0.175 (*)	−0.959 (**)
GGHE-D <- GDP		−0.683(**)	-	−0.683(**)
IMR <- MMR		0.156 (*)	-	0.156 (*)
IMR <- BCGI		−0.0041 (0.774)	-	−0.0041 (0.774)
IMR <- FR		1.032 (**)	0.136 (*)	1.168 (*)
IMR <- GGHE-D		−0.0012 (0.915)	0.308 (**)	0.306 (*)
IMR <- GDP		0.191 (**)	−1.126 (**)	−0.941 (**)
IMR <- OOP		-	−0.012 (*)	−0.012 (**)

** significant at the 1 percent level and * significant at 5 percent level.

**Table 4:** The fitted covariances of observed variables (standardised) for each factor of the causality of infant mortality in Ethiopia, (2000–2019).

	MMR	BCGI	IMR	GGHE-D	FR	OOP	GDP
MMR	1						
BCGI	−0.75	1					
IMR	0.99	−0.73	1				
GGHE-D	0.79	−0.79	0.81	1			
FR	0.99	−0.72	0.99	0.80	1		
OOP	−0.17	0.39	−0.11	−0.07	−0.09	1	
GDP	−0.96	0.66	−0.95	−0.69	−0.96	0.09	1

**Table 5:** The equation-level goodness of fit for the causality of infant mortality in Ethiopia with unstandardised residuals, (2000–2019).

Observed Variables	Variance			R-Squared	mc	mc2
	Fitted	Predicted	Residual			
MMR	49,877.49	49,500.85	376.65	0.993	0.996	0.993
BCGI	36.63	27.65	8.98	0.755	0.869	0.755
IMR	244.65	244.51	0.14	0.999	0.996	0.999
GGHE-D	0.276	0.13	0.15	0.466	0.682	0.466
FR	0.544	0.52	0.025	0.955	0.978	0.955
Overall				0.995		

mc = correlation between depvar and its prediction. mc2 = mc^2^ is the Bentler-Raykov squared multiple correlation coefficient.

**Table 6:** The covariance residuals for each factor of the association of infant mortality in Ethiopia, (2000–2019).

	MMR	BCGI	IMR	GGHE-D	FR	OOP	GDP
MMR	0.076						
BCGI	0.446	0.515					
IMR	0.047	−0.445	0.016				
GGHE-D	0.113	−0.472	0.020	0.000			
FR	0.037	−0.370	0.007	0.000	0.000		
OOP	0.731	1.034	−0.818	−1.841	−0.716	0.000	
GDP	0.022	0.000	−0.003	−0.000	−0.000	0.000	0.000

**Table 7:** The finalized and accepted structural equation model for the infant mortality rate in Ethiopia (from 2000–2019).

	Coef.	Std. Err.	z	*p* > |z|	[95% Conf. Interval]	
GGHE-D <-						
GDP	−0.6819305	0.1196231	−5.70	0.000	−0.9163875	−0.4474736
_cons	3.343989	0.4232345	7.90	0.000	2.514464	4.173513
FR <-						
GGHE-D	0.2547491	0.071664	3.55	0.000	0.1142903	0.3952079
GDP	−0.7855652	0.0623844	−12.59	0.000	−0.9078363	−0.663294
_cons	7.893804	1.260267	6.26	0.000	5.423727	10.36388
BCGI <-						
GGHE-D	−0.6394371	0.1900197	−3.37	0.001	−1.011869	−0.2670054
OOP	0.3266416	0.1577665	2.07	0.038	0.0174249	0.6358584
GDP	0.1888563	0.1671475	1.13	0.259	−0.1387468	0.5164595
_cons	8.858733	2.392873	3.70	0.000	4.168789	13.54868
MMR <-						
GGHE-D	0.246499	0.0531217	0.62	0.385	−0.0710667	0.1371665
FR	0.9609476	0.0323262	29.73	0.000	0.8975893	1.024306
OOP	−0.0707044	0.0289071	−2.45	0.014	−0.1273614	−0.0140475
_cons	−3.269208	0.5745308	−5.69	0.000	4.395268	−2.143148
IMR <-						
MMR	0.1454458	0.0593495	2.62	0.009	0.0391229	0.2717687
BCGI	−0.0071356	0.0144006	−0.29	0.774	−0.0323602	0.024089
GGHE-D	−0.002406	0.0420536	−0.03	0.976	−0.0836643	0.081183
FR	1.025088	0.0543037	18.88	0.000	0.9186548	1.131521
GDP	0.1912811	0.0313778	6.10	0.000	0.1297818	0.2527805
_cons	−4.382065	0.7871316	−5.57	0.000	−5.924815	−2.839315
mean (OOP)	10.6367	1.696609	6.27	0.000	7.311404	13.96199
mean(GDP)	1.612898	0.3391696	4.76	0.000	0.9481377	2.277658
var(e. GGHE-D)	0.5349707	0.1631493			0.2942661	0.9725676
var(e.FR)	0.0450516	0.0196886			0.01913	0.1060976
var(e.BCGI)	0.2451441	0.0921224			0.1173679	0.5120277
var(e.MMR)	0.0076	0.0033638				
var(e.IMR)	0.0005578	0.0002494			0.0002322	0.0013399
var (OOP)	1					
var (GDP)	1					
cov(OOP,GDP)	0.095317	0.2215753	0.43	0.667	−0.3389626	0.5295965

## Data Availability

“World Bank Open Data|Data”. Retrieved 4 October 2022 (https://data.worldbank.org/).
